# Serum indoxyl sulfate concentrations associate with progression of chronic kidney disease in children

**DOI:** 10.1371/journal.pone.0240446

**Published:** 2020-10-27

**Authors:** Johannes Holle, Marietta Kirchner, Jürgen Okun, Aysun K. Bayazit, Lukasz Obrycki, Nur Canpolat, Ipek Kaplan Bulut, Karolis Azukaitis, Ali Duzova, Bruno Ranchin, Rukshana Shroff, Cengiz Candan, Jun Oh, Günter Klaus, Francesca Lugani, Charlotte Gimpel, Rainer Büscher, Alev Yilmaz, Esra Baskin, Hakan Erdogan, Ariane Zaloszyc, Gül Özcelik, Dorota Drozdz, Augustina Jankauskiene, Francois Nobili, Anette Melk, Uwe Querfeld, Franz Schaefer, on behalf of the 4C Study Consortium

**Affiliations:** 1 Department of Pediatric Gastroenterology, Nephrology and Metabolic Diseases, Charité –Universitätsmedizin Berlin, Berlin, Germany; 2 Institute of Medical Biometry and Informatics, University of Heidelberg, Heidelberg, Germany; 3 Department of General Pediatrics, Division of Inherited Metabolic Diseases, Center of Pediatric and Adolescent Medicine, University Hospital Heidelberg, Heidelberg, Germany; 4 Department of Pediatric Nephrology, Cukurova University School of Medicine, Adana, Turkey; 5 Department of Nephrology, Kidney Transplantation and Hypertension, The Children`s Memorial Health Institute, Warsaw, Poland; 6 Division of Pediatric Nephrology, Istanbul University Cerrahpasa Faculty of Medicine, Istanbul, Turkey; 7 Department of Pediatric Nephrology, Ege University Faculty of Medicine, Izmir, Turkey; 8 Clinic of Pediatrics, Institute of Clinical Medicine, Vilnius University, Vilnius, Lithuania; 9 Division of Pediatric Nephrology, Hacettepe University Faculty of Medicine, Ankara, Turkey; 10 Pediatric Nephrology Unit, Hôpital Femme Mere Enfant, Hospices Civils de Lyon, Bron, France; 11 Division of Pediatric Nephrology, Great Ormond Street Hospital for Children, London, United Kingdom; 12 Pediatric Nephrology, Göztepe Educational and Research Hospital, Istanbul, Turkey; 13 Pediatric Nephrology, UKE University Children´s Hospital, Hamburg, Germany; 14 Pediatric Nephrology, KfH and University of Marburg, Marburg, Germany; 15 Pediatric Nephrology, Istituto Giannina Gaslini, Genova, Italy; 16 Department of Internal Medicine IV, University Medical Center & Faculty of Medicine–University of Freiburg, Breisgau, Germany; 17 Pediatric Nephrology, University Children´s Hospital, Essen, Germany; 18 Pediatric Nephrology, Istanbul Medical Faculty, Istanbul, Turkey; 19 Pediatric Nephrology, Baskent University Faculty of Medicine, Ankara, Turkey; 20 Department of Pediatric Nephrology, Bursa Yuksek Ihtisas Training and Research Hospital, Bursa, Turkey; 21 Division of Pediatric Nephrology, Hopital de Hautepierre, Strasbourg, France; 22 Pediatric Nephrology, Sisli Hamidiye Etfal Training and Research Hospital, Istanbul, Turkey; 23 Department of Pediatric Nephrology and Hypertension, Jagiellonian University Medical College, Krakow, Poland; 24 Service de Pédiatrie 2, Centre Hospitalier Universitaire de Besançon, Besancon, France; 25 Pediatric Nephrology, Hannover Medical School, Hannover, Germany; 26 Division of Pediatric Nephrology, Center of Pediatric and Adolescent Medicine, University Hospital Heidelberg, Heidelberg, Germany; International University of Health and Welfare, School of Medicine, JAPAN

## Abstract

The uremic toxins indoxyl sulfate (IS) and p-cresyl sulfate (pCS) accumulate in patients with chronic kidney disease (CKD) as a consequence of altered gut microbiota metabolism and a decline in renal excretion. Despite of solid experimental evidence for nephrotoxic effects, the impact of uremic toxins on the progression of CKD has not been investigated in representative patient cohorts. In this analysis, IS and pCS serum concentrations were measured in 604 pediatric participants (mean eGFR of 27 ± 11 ml/min/1.73m2) at enrolment into the prospective Cardiovascular Comorbidity in Children with CKD study. Associations with progression of CKD were analyzed by Kaplan-Meier analyses and Cox proportional hazard models. During a median follow up time of 2.2 years (IQR 4.3–0.8 years), the composite renal survival endpoint, defined as 50% loss of eGFR, or eGFR <10ml/min/1.73m2 or start of renal replacement therapy, was reached by 360 patients (60%). Median survival time was shorter in patients with IS and pCS levels in the highest versus lowest quartile for both IS (1.5 years, 95%CI [1.1,2.0] versus 6.0 years, 95%CI [5.0,8.4]) and pCS (1.8 years, 95%CI [1.5,2.8] versus 4.4 years, 95%CI [3.4,6.0]). Multivariable Cox regression disclosed a significant association of IS, but not pCS, with renal survival, which was independent of other risk factors including baseline eGFR, proteinuria and blood pressure. In this exploratory analysis we provide the first data showing a significant association of IS, but not pCS serum concentrations with the progression of CKD in children, independent of other known risk factors. In the absence of comorbidities, which interfere with serum levels of uremic toxins, such as diabetes, obesity and metabolic syndrome, these results highlight the important role of uremic toxins and accentuate the unmet need of effective elimination strategies to lower the uremic toxin burden and abate progression of CKD.

## Introduction

Identifying modifiable risk factors for the continuous loss of renal function that characterizes chronic kidney disease (CKD) is a major challenge towards improving treatment for patients with CKD. While hypertension and proteinuria are established risk factors for progression of CKD both in adults and children, even optimal current treatment still cannot prevent many patients progressing to end-stage kidney disease [1–3].

CKD leads to alterations in the gut microbiome (dysbiosis) and accumulation of gut derived uremic toxins in plasma [4]. We have previously shown that the uremic toxins indoxyl sulfate (IS) and p-cresyl sulfate (pCS) are inversely correlated to the estimated glomerular filtration rate (eGFR) in children and adolescents across all stages of CKD [5].

The accumulation of these toxins in the circulation has deleterious consequences, as their plasma concentrations are associated with cardiovascular events in adult CKD patients and with surrogate markers of cardiovascular disease (CVD) in pediatric patients [5, 6]. In addition, uremic toxins may affect kidney cells directly and thus promote progression of CKD. IS and pCS have direct toxic effects on tubular epithelial cells *in vitro* [7]. In animal models the accumulation of these toxins in renal tubular cells has been demonstrated to cause direct cytotoxicity, tubulointerstitial fibrosis and acceleration of CKD progression [8, 9]. While there is experimental evidence both *in vitro* and *in vivo* for nephrotoxic effects of uremic toxins, associations of IS and pCS serum levels with progression of CKD have only been investigated in a single study of adult CKD patients to date [10]. Here we investigated the impact of IS and pCS on progression of CKD in a cohort of more than 600 children with CKD of the prospective Cardiovascular Comorbidity in Children with Chronic Kidney Disease (4C) study.

## Materials and methods

### Study design

The 4C study is a prospective observational cohort study conducted at 55 pediatric nephrology centers in 12 European countries. Children with an initial eGFR of 10–60 ml/min per 1.73m^2^ were included in the study. Written informed consent was obtained from all patients and/or parents. The study was approved by the Ethics Board of the University of Heidelberg (S-032/2009) and subsequently by the local review boards of each participating institution (for a complete list of review boards, see [11]). All procedures performed involving human participants were in accordance with the Declaration of Helsinki. Additional details, including exclusion criteria, have been published in detail previously [12].

In the 4C study all participating centers were visited annually by trained regional coordinators. Every 6 months an update of the medical history and medication, clinical status and anthropometric data were obtained and blood and urine samples were collected and stored for central laboratory analysis. Office systolic blood pressure (SBP) was documented as an average of three oscillometric measurements using local devices. SBP and body mass index (BMI) were normalized for height and age (SBP SDS and BMI SDS respectively) as previously described. [12]. Glomerular filtration rate was estimated (eGFR) using the 2009 bedside Schwartz formula [13]. Patients were categorized according to their country of residency as Mediterranean (Turkey, Portugal, France and Italy) and non-Mediterranean (Germany, Austria, Switzerland, Poland, Lithuania, Serbia, Czech Republic and United Kingdom). Renal diagnoses were grouped into congenital anomalies of the kidney and urinary tract (CAKUT), tubulointerstitial disorders, glomerulopathies, chronic kidney disease after acute kidney injury (post-AKI CKD) and others.

### Laboratory methods

Serum samples were available in 604 patients at the baseline visit. Total serum levels of IS and pCS were measured centrally using reverse-phase separation and fluorescence detection, as described previously [5].

### Statistical analyses

Baseline characteristics are given as mean ± standard deviation (SD), median with interquartile range (IQR) or frequencies (n, %), as appropriate. Serum concentrations of IS and pCS measured at the baseline visit were used to analyze an association with renal survival time during follow up. Summary statistics stratified by CKD stage are provided for IS and pCS. As the distribution of IS and pCS was skewed, these variables were log transformed for further analysis. The assumption of normal distributed data was proven (visual inspection and comprehensive summary statistic) after log transformation. Correlation of IS and pCS (log transformed) with eGFR was quantified by means of Pearson correlation coefficient. In a multivariable linear regression analysis the association of log transformed IS (pCS) with clinically relevant variables at baseline was assessed [5].

The primary endpoint was renal survival defined as time from baseline to the composite event of either eGFR of <10ml/min or 50% loss in eGFR or start of RRT, whatever occurred first, as described previously [14, 15]; if eGFR<10 or 50% loss in eGFR occurred between two visits, linear interpolation was used to determine the “exact” point in time. As sensitivity analyses, two procedures were applied to prove consistency of our results because of not having observed the precise date of the event. First, instead of applying linear interpolation we used the later of the two visits as occurrence of the event. Second, subjects were interval-censored instead of linear interpolation. In the first case, analyses as described below can just be repeated as only the event time for some subjects changed. In the second case ICPHREG SAS procedure for interval-censored data was applied to the data with information on the two points between which the event occured. All data were administratively censored as of June 2019. Early drop-outs were censored at last contact and reasons for drop-out are summarized. Kaplan-Meier curves stratified by quartiles of IS or pCS levels are presented with *p* value of the log-rank test and median survival time. By means of a Cox proportional hazards model the association of IS or pCS with renal survival was analyzed providing adjusted hazard ratio (HR) with 95% confidence interval (CI) and *p* value. Different models were considered to evaluate if adding IS or pCS to the standard model (model 0) improved model fit. Covariates of model 0 were sex, renal diagnoses, residency, and baseline levels of eGFR, age, proteinuria (log transformed), SBP SDS, BMI SDS, serum albumin, hemoglobin and phosphorus. In model 1, IS or pCS was added to the set of basic covariates. By means of a forest plot the effect (HR with 95% CI) of IS (pCS) on renal survival was visualized for different patient characteristics (e.g. sex: male vs. female) based on separate Cox models with IS (pCS), the respective patient characteristic and the other remaining covariates included. Model 1 was repeated with categorized IS or pCS (quartile groups, compare Kaplan-Meier curves). Quality of the models was compared based on the Akaike information criterion (AIC), with lower values indicating a better fit. The AIC value is the expected, relative distance between the fitted model and the unknown true mechanism that generated the observed data.

A subset of patients with very high IS levels was identified by determining the upper 95% prediction limit (UPL) in a regression of eGFR on log-transformed IS (S1 Fig) ^5^. All analyses with respect to IS were carried out separately for the whole sample (total cohort) and, as sensitivity analyses, for the sample excluding patients with IS levels above the 95% UPL (consolidated cohort). As this was an exploratory study, p-values were interpreted descriptively and *p*<0.05 was considered statistically significant. Missing values were not imputed. Data were analyzed using SAS® Software version 9.4 (SAS Inc. Cary/ NC, USA).

## Results

### Patient characteristics

Between October 2009 and August 2011 a total of 704 children were enrolled in the 4C study. IS and pCS serum levels were measured in 604 patients with available blood samples and an eGFR between 10–60ml/min per 1.73 m^2^ at baseline denoted as total cohort in the following. Patient characteristics are given in Table 1; these are comparable to those in the entire 4C cohort [16]. Mean age was 12.1 ± 3.3 years with a mean eGFR of 27 ± 11 ml/min per 1.73 m^2^. CAKUT was the most common (70%) underlying primary renal disease.

**Table 1:** Patient characteristics.

Age (years)	12.1 ± 3.3	n = 604
Male sex	399 (66%)	n = 604
Diagnosis		n = 604
CAKUT	420 (70%)	
Glomerulopathy	49 (8%)	
Post-AKI	27 (5%)	
Tubulointerstitial	82 (14%)	
Others	26 (4%)	
Country		n = 604
GER, CH, AT	118 (20%)	
FR, IT, PT	97 (16%)	
PL, LT, CZ, SRB	53 (9%)	
UK	35 (6%)	
Turkey	301 (50%)	
BMI SDS	0.08 ± 1.30	n = 604
Systolic blood pressure SDS	0.81 ± 1.37	n = 604
eGFR (ml/min)	26.6 ± 11.2	n = 604
uPCr (mg/mg)	1.3 (2.6)	n = 579
Serum Albumin (g/l)	39.0 ± 6.30	n = 604
Serum phosphorus (mmol/l)	1.55 ± 0.38	n = 604
Hemoglobin (g/l)	11.6 ± 1.65	n = 594

Data shown as mean ± standard deviation, median (interquartile range) or n (%) as appropriate. CAKUT = congenital anomalies of the kidney and urinary tract; Post-AKI = chronic kidney disease after acute kidney injury; BMI = body mass index; eGFR = estimated glomerular filtration rate; uPCr = Urinary protein creatinine ratio; SDS = standard deviation score. Countries: GER = Germany; CH = Switzerland; AT = Austria; FR = France; IT = Italy; PT = Portugal; PL = Poland; LT = Lithuania; CZ = Czech Republic; SRB = Serbia; UK = United Kingdom.

### Measurement of indoxyl sulfate and p-cresyl sulfate

Serum IS and pCS concentrations were negatively correlated with eGFR (r = -0.46 for IS and r = -0.43 for pCS respectively). In 45 patients IS levels exceeded the 95% UPL calculated for the total cohort. These patients had a younger age (11.2 ± 3.3 versus 12.2 ± 3.3 years) and a lower hemoglobin (11.1 ± 1.70 versus 11.7 ± 1.64 g/l) compared to the rest of the cohort, but no other significant differences in baseline characteristics including medication with phosphate binders or antibiotics and iron supplementation (S1 Table). Additional sensitivity analyses were performed excluding these patients with IS levels exceeding the 95% UPL (denoted as consolidated cohort, S1 Fig and Table 2).

**Table 2:** Serum levels of indoxyl sulfate and p-cresyl sulfate according to CKD stage.

	Total cohort	CKD 3a	CKD 3b	CKD 4	CKD 5	Consolidated cohort	Excluded patients
Patients (n)	604	41	174	292	97	559	45
IS mean (SD)	25.5 (86.4)	4.00 (11.7)	17.8 (65.6)	31.4 (105)	30.8 (78.3)	7.79 (9.95)	246 (218)
IS median (IQR)	5.30 (8.70)	1.40 (1.60)	3.45 (3.70)	6.25 (7.30)	13.0 (11.0)	5.00 (6.80)	178 (113)
IS quartiles							
• <2.9	152 (25%)	35 (85%)	74 (43%)	39 (13%)	4 (4%)	152 (27%)	0 (0%)
• 2.9–5.3	152 (25%)	4 (10%)	58 (33%)	85 (29%)	5 (5%)	152 (27%)	0 (0%)
• 5.3–11.6	151 (25%)	0 (0.0%)	24 (14%)	98 (33%)	29 (30%)	151 (27%)	0 (0%)
• >11.6	149 (25%)	2 (5%)	18 (10%)	70 (24%)	59 (61%)	104 (19%)	45 (100%)
Patients (n)	604	41	174	292	97	604	0
pCS mean (SD)	21.1 (18)	6.99 (6)	13.5 (11.2)	22.4 (16.1)	36.7 (22)	21.1 (18)	
pCS median (IQR)	17.2 (22)	6.20 (7)	9.80 (15.2)	19.7 (19.7)	34.1 (30)	17.2 (22)	
pCS quartiles							
• <7.9	153 (25%)	26 (63%)	67 (39%)	49 (17%)	11 (11%)	153 (25%)	
• 7.9–17.2	149 (25%)	13 (32%)	53 (31%)	74 (25%)	9 (9%)	149 (25%)	
• 17.2–29.6	151 (25%)	1 (2%)	41 (24%)	92 (32%)	17 (18%)	151 (25%)	
• >29.6	151 (25%)	1 (2%)	13 (8%)	77 (26%)	60 (62%)	151 (25%)	

Serum levels of indoxyl sulfate (IS) and p-cresyl sulfate (pCS) in the total cohort and across CKD stage 3–5 at study entry. For survival analyses, patients were grouped according to IS and pCS quartiles respectively. In the consolidated cohort, patients with IS levels > 95% of the upper prediction limit were excluded. IS and pCS serum levels in μmol/l. CKD = chronic kidney disease; IS = indoxyl sulfate; pCS = p-cresyl sulfate; SD = standard deviation; IQR = interquartile range.

In a multivariable regression analysis (S2 Table) IS levels showed positive associations with urea (p = 0.004) and negative associations with eGFR (p<0.001) and uric acid (p<0.001). Age, sex, diagnosis, residency, physical activity, BMI SDS, serum phosphorus, serum albumin, urinary protein/creatinine ratio and treatment with calcium-based phosphate binders, iron or antibiotics were not associated with IS serum levels. pCS levels were positively associated with age (p = 0.008), non-Mediterranean residency (p<0.001), serum albumin (p<0.001), urea (p = 0.038) and iron therapy (p = 0.034) and negatively associated with the diagnosis of glomerulopathies (p = 0.008), eGFR (p<0.001), uric acid (p = 0.002) and physical activity (> 4 hours per week, p = 0.044).

### Progression of CKD

**Fig 1:**
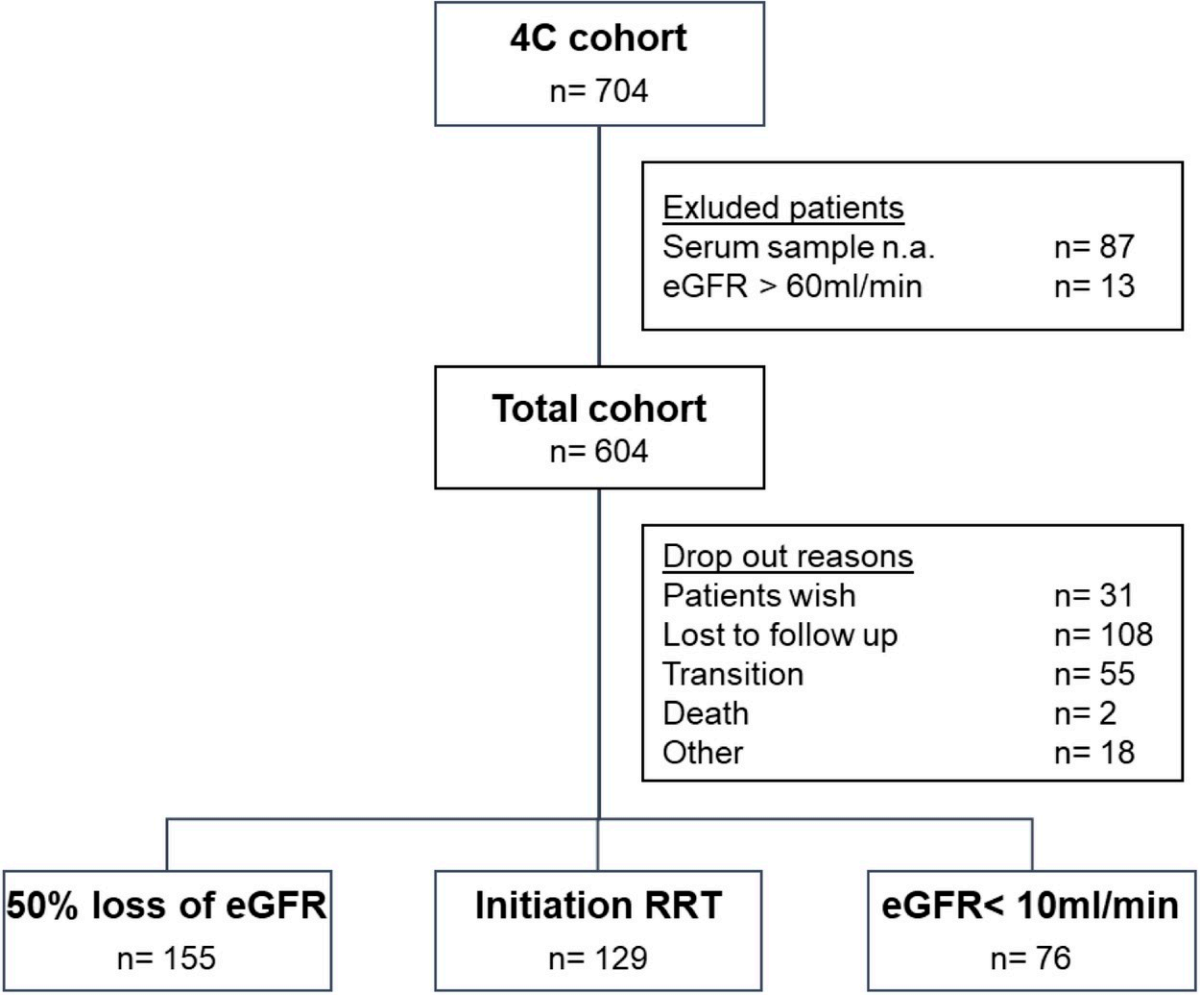
Study description with progression to primary endpoint and reasons for drop out. eGFR = estimated glomerular filtration rate; RRT = renal replacement therapy.

During a median follow up time of 2.2 years (IQR 4.3–0.8 years, maximum follow up 8.8 years), the composite renal survival endpoint was reached by 360 patients (59.6%). The median renal survival time was 3.5 years (95% CI, 2.9–3.9). Frequency and allocation of different endpoints as well as drop-out reasons are shown in Fig 1.

### Association of IS and pCS with progression of CKD

**Fig 2:**
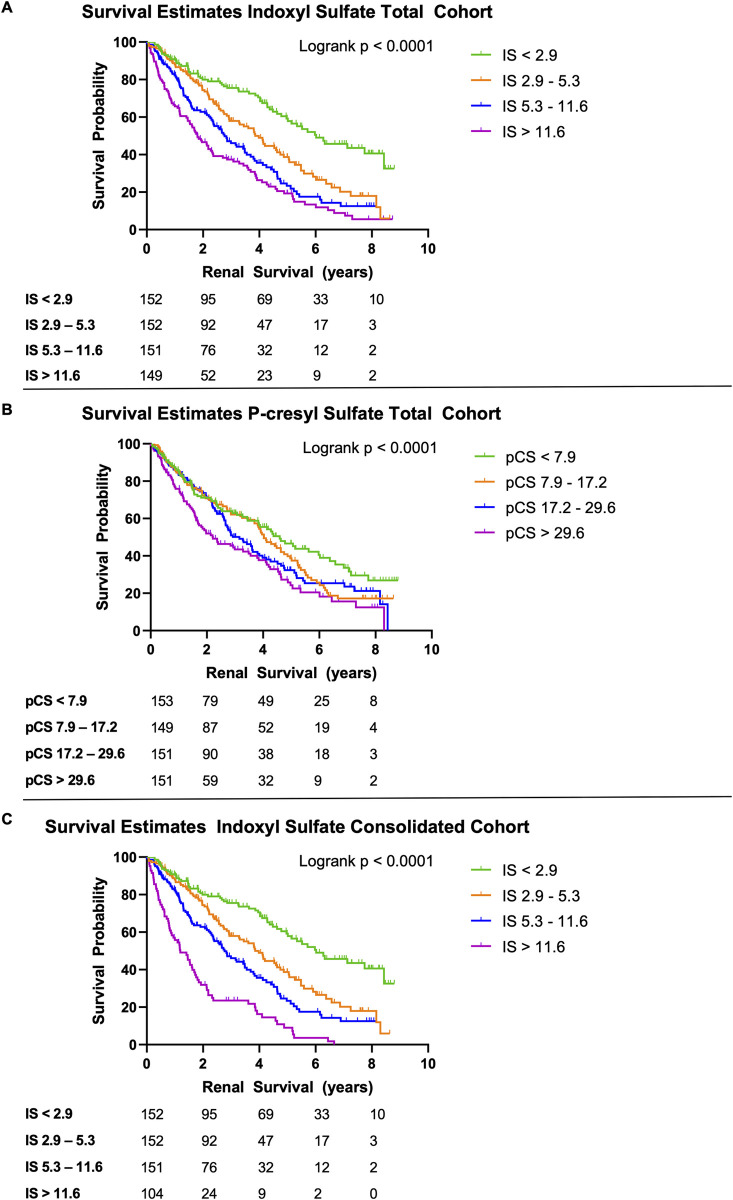
Renal survival in children with CKD according to quartiles of serum indoxyl sulfate (panels A, C) and p-cresyl sulfate (panel B). Results for total cohort (n = 604) are given in panels A and B. Panel C shows renal survival analysis by indoxyl sulfate quartiles after exclusion of patients with serum indoxyl sulfate above the 95% upper prediction limit (consolidated cohort, n = 559). Indoxyl sulfate and p-cresyl sulfate serum levels are given in μmol/l. Q1-4 indicate distribution quartiles.

We found a significant association of serum IS and pCS concentrations with renal survival (p<0.0001, Fig 2A and 2B). Across all patients, the median survival time was lower in patients with IS and pCS levels in the 4th quartile (1.5 years, 95% CI [1.1, 2.0] for IS and 1.8 years, 95%CI [1.5, 2.8] for pCS) compared to those patients in the 1st quartile (6.0 years, 95% CI [5.0, 8.4] and 4.4 years, 95% CI [3.4, 6.0] for IS and pCS respectively). Exclusion of patients with IS levels above the 95% UPL (consolidated cohort), resulted in an even shorter median survival time (Fig 2C, 0.9 years, 95% CI [0.6, 1.5] of patients with IS levels in the 4th quartile).

In a multivariable Cox regression model, older age, male sex, diagnosis of tubulointerstitial disease or post-AKI CKD as well as the presence of proteinuria, higher systolic blood pressure, lower baseline eGFR, higher serum phosphorus, lower serum albumin and hemoglobin levels contributed to a higher likelihood of reaching the composite endpoint (Table 3 and Model 0, total cohort, Table 4). The levels of IS, but not pCS, contributed significantly to progression of CKD (Table 4). In detail, we found a significant association of IS levels in the 4th quartile compared to the 1st quartile and renal survival (Model 1b). Furthermore, after exclusion of patients with IS levels >95% UPL, IS as a continuous variable (Model 1e) was significantly associated with renal survival. The effect on renal survival of IS levels in the 4th quartile compared to the 1st quartile was even higher in patients of the consolidated cohort compared to patients of the total cohort (Model 1f, HR 1.94, 95% CI [1.29, 2.93] versus Model 1b, HR 1.62, 95% CI [1.12, 2.35]). Similar results were obtained for the additional sensitivity analyses without linear interpolation where the precise date of the event was not observed by taking the date of the later visit (S3 Table) or by considering interval censoring (S4 Table).

**Table 3:** Multivariable Cox regression model of variables associated with renal survival without indoxyl sulfate and p-cresyl sulfate (model 0, total cohort).

Variable	Estimate	Standard Error	p-value	Hazard Ratio	95% Hazard Ratio Confidence Intervall	
Female sex	-0.24	0.12	0.049	0.79	0.62	0.10
Age (years)	**0.08**	**0.02**	**< .001**	**1.09**	**1.05**	**1.13**
Diagnosis						
Glomerulopathies	0.24	0.22	0.261	1.28	0.83	1.96
Post-AKI	**0.85**	**0.27**	**0.001**	**2.34**	**1.40**	**3.91**
Other	0.18	0.30	0.558	1.19	0.66	2.16
Tubulointerstitial	**0.74**	**0.16**	**< .001**	**2.10**	**1.54**	**2.87**
Non-Mediterranean	0.12	0.12	0.309	1.13	0.89	1.43
BMI SDS	-0.02	0.05	0.682	0.98	0.90	1.07
Systolic BP SDS	**0.15**	**0.04**	**0.001**	**1.17**	**1.08**	**1.26**
eGFR (ml/min/1.73m^2^)	**-0.08**	**0.01**	**< .001**	**0.93**	**0.91**	**0.94**
uPCr (mg/mg)	**0.34**	**0.06**	**< .001**	**1.40**	**1.24**	**1.57**
Hemoglobin (g/l)	**-0.14**	**0.04**	**< .001**	**0.87**	**0.81**	**0.94**
Serum phosphorus (mmol/l)	**0.39**	**0.15**	**0.010**	**1.47**	**1.10**	**1.98**
Serum albumin (g/l)	**-0.06**	**0.01**	**< .001**	**0.94**	**0.92**	**0.96**

Cox Model for the total cohort with variables measured at study entry (n = 569 patients). Diagnostic groups were compared to patients with the diagnosis of CAKUT. Non-Mediterranean residency was compared to patients living in Mediterranean countries. CAKUT = congenital anomalies of the kidney and urinary tract; Post-AKI = chronic kidney disease after acute kidney injury; BMI = body mass index; eGFR = estimated glomerular filtration rate; uPCr = Urinary protein creatinine ratio; SDS = standard deviation score.

**Table 4:** Multivariable Cox proportional hazard analysis for the association of IS and pCS serum levels with renal survival.

	AIC	Parameter	Estimate	Standard Error	p-value	Hazard Ratio	95% Hazard Ratio Confidence Intervall
**Model 0 total cohort**	3412.5	---				---	---	---
Model 1a	3412.2	Log IS	0.07	0.04	0.131	1.07	0.98	1.17
Model 1b	3400.3	Q2: 2.9–5.3	0.15	0.18	0.426	1.16	0.80	1.65
		Q3: 5.3–11.6	-0.15	0.19	0.422	0.86	0.59	1.25
		Q4: >11.6	**0.48**	**0.19**	**0.011**	**1.62**	**1.12**	**2.35**
Model 1c	3414.1	Log pCS	-0.037	0.06	0.533	0.96	0.86	1.08
Model 1d	3416.7	Q2: 7.9–17.2	-0.22	0.17	0.201	0.80	0.57	1.12
		Q3: 17.2–29.6	-0.17	0.18	0.317	0.84	0.60	1.18
		Q4: >29.6	-0.13	0.18	0.464	0.88	0.61	1.25
**Model 0 consolidated cohort**	3088.5	---				---	---	---
Model 1e	3083.9	Log IS	**0.20**	**0.08**	**0.001**	**1.23**	**1.05**	**1.43**
Model 1f	3072.4	Q2: 2.9–5.3	0.17	0.18	0.371	1.18	0.82	1.69
		Q3: 5.3–11.6	-0.10	0.20	0.600	0.90	0.62	1.32
		Q4: >11.6	**0.66**	**0.21**	**0.002**	**1.94**	**1.29**	**2.93**

Model 0 represents a Cox model with sex, age, diagnosis, region of residence, body mass index SDS, blood pressure SDS, estimated glomerular filtration rate, urinary protein creatinine ratio, hemoglobin, serum phosphorus and albumin as covariates, calculated either for the total cohort (n = 569) or for the consolidated cohort (excluding patients with IS levels exceeding the 95% upper prediction limit, n = 526). Model 1a includes serum IS, 1b IS quartiles, 1c serum pCS and 1d serum pCS quartiles as a covariate to model 0. Models 1e and 1 f include serum IS and serum IS quartiles respectively as covariates to model 0 in the consolidated cohort. IS and pCS are used as logarithmic values in all models. For all variables associated with renal survival (model 0, total cohort) please refer to Table 3. IS = indoxyl sulfate; pCS = p-cresyl sulfate; Q = quartiles of serum IS and pCS levels, respectively; AIC = Akaike information criterion.

**Fig 3:**
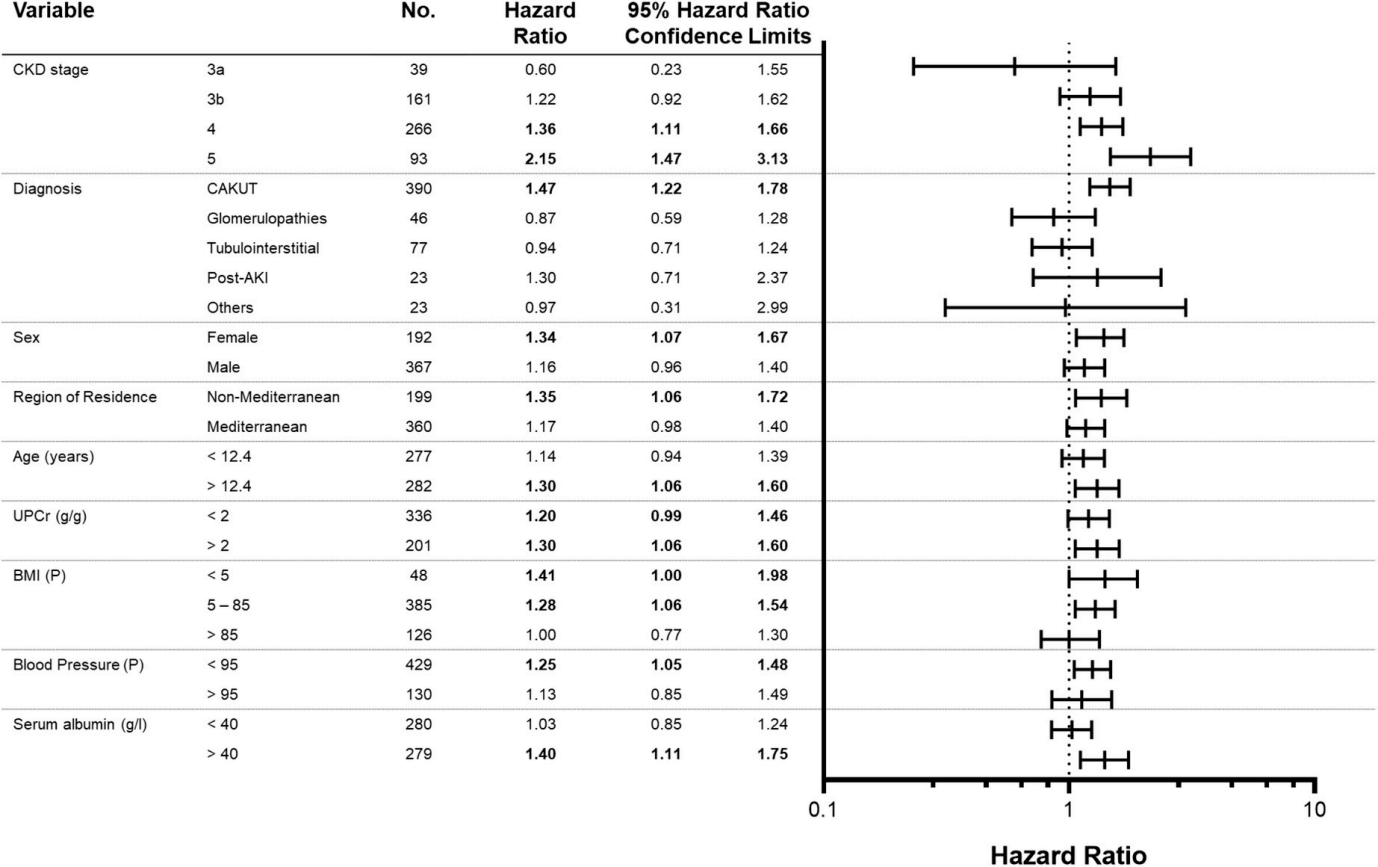
Excess renal survival risk attributable to high versus low serum indoxyl sulfate levels according to patient characteristics of the consolidated cohort. Each model was adjusted for all other covariates. Hazard ratio and 95% Confidence Limits indicate significance of interaction between IS status and respective patient characteristic. The median split of indoxyl sulfate was at 5.3 μmol/l. P = percentile; UPCr = urinary protein creatinine ratio.

For the consolidated cohort, the effect of IS on renal survival in the different subsets adjusted for the other covariates is presented in Fig 3 by means of a forest plot. We found an interaction of IS levels with baseline CKD stage on renal survival, the highest hazard ratio being present in patients with CKD stage 5 at enrolment. Furthermore, the excess risk attributable to high IS concentrations was significantly elevated in normal-weight and normotensive, female and older patients, patients with CAKUT and those with non-Mediterranean residency.

## Discussion

In this exploratory analysis we provide the first data showing the impact of microbial derived uremic toxins on the progression of CKD in a representative multicenter cohort of children with CKD. We found a significant association of serum IS concentrations with the prospective progression of CKD, independent of other known traditional risk factors, i.e. baseline eGFR, proteinuria and blood pressure. By contrast the pCS concentrations showed no association with renal survival after adjustment for baseline eGFR.

There is a growing body of evidence on how IS might contribute to the progression of CKD. *In vitro*, IS was shown to have direct toxic effects on tubular cells leading to increased levels of oxidative stress, fibrosis and inflammation [7, 17]. In animal models, IS seems to accelerate CKD progression by nephrotoxic effects [17]. Furthermore, vascular effects of IS might also contribute to progression of CKD. IS promotes vascular calcification [18] and inflammation [19] *in vitro* and in animal models of CKD [20]. Moreover, serum IS levels are associated with the surrogate markers of cardiovascular disease (intima media thickness and pulse walve velocity) in children [5] and with diminished endothelial function in adults with CKD [21]. Accordingly, the toxicity of IS *in vitro* and in animal models depends on its concentration, and this is reflected by the fact that those patients with IS levels in the 4th quartile have the highest risk for progression of CKD [22]. In the only clinical study in adult patients investigating the impact of IS and pCS serum levels on progression of CKD [10], 269 patients were followed up for 21 months; only 35 patients progressed to ESKD or had a decline in eGFR of 50% or more. In that group with progressive disease, there was a significant association of both IS and pCS serum concentrations with renal survival. However, almost half of the patients had diabetes, which is known to have a significant impact not only on the progression of CKD but also on the serum levels of uremic toxins [23]. Recently, it has been shown that lower kidney clearance of six endogenous solutes, including IS, but not pCS, are associated with a higher risk of CKD progression in adults participating in the CRIC study [24], which is in line with our results. In our study we provide the first results about the association of IS with progression of CKD in pediatric patients who had no other comorbidities which might interfere with renal survival. This allows to attribute the effect on renal survival more directly to serum concentrations of uremic toxins. This may explain why we identified a significant association with IS but not with pCS.

Our large study population permitted further statistical analysis of the relative contribution of IS levels to renal survival. The impact of IS levels increased with each stage of CKD, which is most likely due to accumulation of IS with progressive decrease of eGFR [5]. However, IS serum levels also showed interactions with other risk factors for progression. Thus, high IS levels were still associated with a moderately increased hazard ratio for renal progression in the presence of proteinuria, a diagnosis of CAKUT and for older patients. These are interesting findings as we have shown, that diagnosis of CAKUT itself had no direct influence on IS levels (S2 Table). Moreover, antibiotic treatment was more frequent in patients with CAKUT (29% of patients with CAKUT received antibiotic treatment or prophylaxis within six week before recruitment, S5 Table) compared to other patients (p<0.001), but antibiotics were not independently associated with IS levels. Thus the reason for the enhanced effect of IS in patients with CAKUT remains unexplained.

Proteinuria and low serum albumin are well known risk factors for progression of CKD, but neither proteinuria nor serum albumin were independently associated with IS levels. Nevertheless, IS was associated with a slightly higher hazard ratio for progression of CKD in patients with proteinuria, while the effect of IS on progression depending on serum albumin was decreased in hypoalbuminemia. Although we can not provide data about residual diuresis, measurement of free serum toxin concentrations or urinary toxins concentrations, these findings allow speculations about a higher fraction of free and therefore toxic serum concentrations of IS in patients with proteinuria and hypoalbuminemia. For female patients, our data show a lower hazard ratio than for male patients, i.e. better renal survival, but a significantly higher impact of higher serum levels of IS on renal survival.

Interestingly, the impact of higher IS levels on renal survival was stronger in patients with controlled blood pressure than in hypertensive patients; this finding could indicate that better blood pressure control during follow up, which occurred in this cohort (unpublished observation), led to “unmasking” of the effect of higher IS levels. Furthermore, a higher BMI-SDS was associated with better renal survival in the multivariable model, and the relative contribution of high IS levels to renal survival showed the highest hazard ratio in underweight, possibly malnourished patients. While obesity is regarded as an independent risk factor for progression of CKD in adults and children [25, 26], our data indicate the need for further studies regarding the role of uremic toxins in modulating progression of CKD in obese and non-obese patients. Taken together, our findings highlight the impact of elevated IS levels on renal survival and provide new insights into the pathophysiology of CKD progression in the absence of known risk factors such as obesity and arterial hypertension.

Therapeutic efforts to decelerate progression of CKD by lowering serum IS levels have so far been unsuccessful. A reduction of IS levels by administration of the oral adsorbent AST-120 had no significant effect on renal survival in clinical trials [27–29]. This might be explained by the complex interplay of nutrition and the gut microbiome, both of which essentially contribute to the balance of beneficial metabolites, e.g. short chain fatty acids (SCFAs), and harmful metabolites, e.g. uremic toxins, in CKD. Removal of uremic toxins by dialysis modalities, including intensified hemodialysis, is insufficient [30]. However, it has been shown that the administration of pre- and probiotics as well as SCFAs has beneficial effects on the gut microbiome and decreases the uremic toxin burden *in vivo* [31, 32]. Animal work has suggested that these effects of pre- and probiotics might also attenuate progression of CKD [33]. Therefore, studies using a combination of different treatment modalities such as microbiome-centered therapies (to restore a protective balance in microbiome composition and metabolism) and effective elimination strategies of uremic toxins are needed.

There are several limitations of our analysis. First, the 4C study was not designed specifically to evaluate the effect of microbial derived uremic toxins, but more general effects on progression of CKD. It is known that serum levels of gut-derived uremic toxins, such as IS and pCS, are potentially confounded by various factors such as age, diabetes, obesity and metabolic syndrome, which have a direct impact on the composition and function of the gut microbiome and hence, on microbial metabolism [34, 35]. Furthermore, antibiotic treatment is supposed to have an influence on serum levels of uremic toxins as well [31, 36]. Wherever possible, these factors have been addressed and included to our multivariable models. Nevertheless, it is unclear why some of our patients had extremely high serum levels of IS, but not pCS, above the 95% UPL. There were no significant differences between these patients and the rest of the cohort regarding baseline characteristics, including the frequency of antibiotic treatment or prophylaxis. We cannot completely rule out any pre-analytical bias in sampling, storage or transport to our central laboratory. However, the association of serum IS levels with renal survival was even stronger after exclusion of these patients. Furthermore, we have no information about symptomatic or asymptomatic gastrointestinal disorders. Nutrition and especially protein intake have an influence on microbial metabolism [37, 38] and have been associated with intra-patient variability, as described previously [39]. Although we have no data about nutrition and dietary intake in the 4C-study, we used Mediterranean residency as a surrogate parameter for nutrition. Furthermore, IS and pCS levels were measured only once at study entry, so we are not able to analyze the impact of nutrition on intra-patient variability in more detail.

Finally, we acknowledge the risk of attrition bias as a consequence of the high drop-out rate during our study follow up, limiting the power of our statistical analysis. On the other hand, the large number of patients enrolled in this study and the choice of a composite endpoint enables us to show a significant association of IS with progression of CKD for the first time in children.

In conclusion, our study shows a significant association of serum IS, but not pCS, with the progression of CKD in children, independent of other known risk factors such as baseline GFR, blood pressure and proteinuria. These results highlight the unmet need of effective elimination strategies to lower the uremic toxin burden, especially in patients with advanced CKD stages, in order to abate the progression of CKD.

## Supporting information

S1 Fig Log-transformed serum levels of indoxyl sulfate (A) and p-cresyl sulfate (B) are correlated with eGFR in 604 children with chronic kidney disease at study entry. Linear regression of log-transformed serum levels of indoxyl sulfate (IS) and p-cresyl sulfate (pCS) showed a significant correlation with eGFR (Pearson correlation coefficient r = -0.46 for IS and r = -0.43 for pCS). 45 patients had IS levels above the 95% upper prediction limit (UPL). While no patients had pCS levels above the 95% UPL, some patients had pCS levels below the 95% lower prediction limit.
(PDF)

S1 Table Patient characteristics of patients with indoxyl sulfate levels exceeding the 95% upper prediction limit compared to the rest of the cohort (consolidated cohort). Data shown as mean ± standard deviation, median (interquartile range) or n (%) as appropriate. p-value indicates group difference between consolidated cohort and those patients with IS levels exceeding the 95% UPL. IS = indoxyl sulfate; UPL = upper prediction limit; CAKUT = congenital anomalies of the kidney and urinary tract; Post-AKI = chronic kidney disease after acute kidney injury; BMI = body mass index; eGFR = estimated glomerular filtration rate; uPCr = Urinary protein creatinine ratio; SDS = standard deviation score; Non-Ca phosphate binders = non calcium containing phosphate binders; Ca phosphate binders = calcium containing phosphate binders. Countries: GER = Germany; CH = Switzerland; AT = Austria; FR = France; IT = Italy; PT = Portugal; PL = Poland; LT = Lithuania; CZ = Czech Republic; SRB = Serbia; UK = United Kingdom.
(PDF)

S2 Table Multivariable linear regression models of variables associated with serum levels of indoxyl sulfate and p-cresyl sulfate in 604 children with CKD at baseline. Diagnostic groups were compared to patients with the diagnosis of CAKUT. Residency was defined as living in Mediterranean or Non-Mediterranean countries. Physical activity was compared to being physically inactive (0 hours). SE = standard error; CAKUT = congenital anomalies of the kidney and urinary tract; Post-AKI = chronic kidney disease after acute kidney injury; BMI = body mass index; eGFR = estimated glomerular filtration rate; Ca-based P binders = calcium-based phosphate binders; uPCr = Urinary protein creatinine ratio; SDS = standard deviation score.
(PDF)

S3 Table Multivariable Cox proportional hazard analysis for the association of IS and pCS serum levels with renal survival (sensitivity analysis by taking the later visit if the event occurred between two visits). Survival analysis using the PHREG SAS procedure. If the event occurred between two visits the later of the two visits was taken as occurrence of the event instead of using linear interpolation. Model 0 represents a Cox model with sex, age, diagnosis, region of residence, body mass index SDS, blood pressure SDS, estimated glomerular filtration rate, urinary protein creatinine ratio, hemoglobin, serum phosphorus and albumin as covariates, calculated either for the total cohort (n = 569) or for the consolidated cohort (excluding patients with IS levels exceeding the 95% upper prediction limit, n = 526). Model 1a includes serum IS, 1b IS quartiles, 1c serum pCS and 1d serum pCS quartiles as a covariate to model 0. Models 1e and 1 f include serum IS and serum IS quartiles respectively as covariates to model 0 in the consolidated cohort. IS and pCS are used as logarithmic values in all models. For all variables associated with renal survival (model 0, total cohort) please refer to Table 3. IS = indoxyl sulfate; pCS = p-cresyl sulfate; Q = quartiles of serum IS and pCS levels, respectively.
(PDF)

S4 Table Multivariable Cox proportional hazard analysis for the association of IS and pCS serum levels with renal survival (sensitivity analysis by considering interval censoring if the event occurred between two visits). Survival analysis for interval-censored data using the ICPHREG SAS procedure. Model 0 represents a Cox model with sex, age, diagnosis, region of residence, body mass index SDS, blood pressure SDS, estimated glomerular filtration rate, urinary protein creatinine ratio, hemoglobin, serum phosphorus and albumin as covariates, calculated either for the total cohort (n = 569) or for the consolidated cohort (excluding patients with IS levels exceeding the 95% upper prediction limit, n = 526). Model 1a includes serum IS, 1b IS quartiles, 1c serum pCS and 1d serum pCS quartiles as a covariate to model 0. Models 1e and 1 f include serum IS and serum IS quartiles respectively as covariates to model 0 in the consolidated cohort. IS and pCS are used as logarithmic values in all models. For all variables associated with renal survival (model 0, total cohort) please refer to Table 3. IS = indoxyl sulfate; pCS = p-cresyl sulfate; Q = quartiles of serum IS and pCS levels, respectively.
(PDF)

S5 Table Antibiotic therapy at baseline and within 6 weeks before baseline stratified by diagnosis group. CAKUT = congenital anomalies of the kidney and urinary tract.
(PDF)
