# Supplementary material for: Serum indoxyl sulfate concentrations associate with progression of chronic kidney disease in children
Source: PLoS One. 2020 Oct 27;15(10):e0240446. doi: 10.1371/journal.pone.0240446 (PMC7591021; doi:10.1371/journal.pone.0240446)
Supplement: S1 Fig — Log-transformed serum levels of indoxyl sulfate (A) and p-cresyl sulfate (B) are correlated with eGFR in 604 children with chronic kidney disease at study entry. Linear regression of log-transformed serum levels of indoxyl sulfate (IS) and p-cresyl sulfate (pCS) showed a significant correlation with eGFR (Pearson correlation coefficient r = -0.46 for IS and r = -0.43 for pCS). 45 patients had IS levels above the 95% upper prediction limit (UPL). While no patients had pCS levels above the 95% UPL, some patients had pCS levels below the 95% lower prediction limit. (PDF) [file pone.0240446.s001.pdf]

S1 Fig. Log-transformed serum levels of indoxyl sulfate (A) and p-cresyl sulfate (B) are correlated with eGFR in 604 children with chronic kidney disease at study entry.

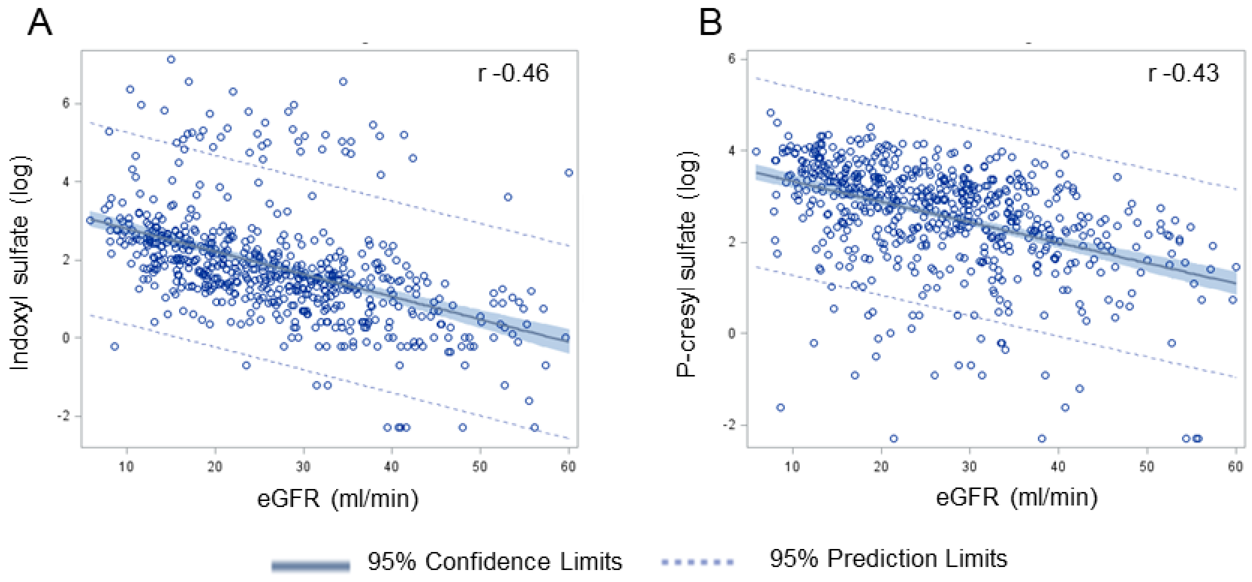

Linear regression of log-transformed serum levels of indoxyl sulfate (IS) and p-cresyl sulfate (pCS) showed a significant correlation with eGFR (Pearson correlation coefficient r= -0.46 for IS and r= -0.43 for pCS). 45 patients had IS levels above the 95% upper prediction limit (UPL). While no patients had pCS levels above the 95% UPL, some patients had pCS levels below the 95% lower prediction limit.
