# Supplementary material for: Serum indoxyl sulfate concentrations associate with progression of chronic kidney disease in children
Source: PLoS One. 2020 Oct 27;15(10):e0240446. doi: 10.1371/journal.pone.0240446 (PMC7591021; doi:10.1371/journal.pone.0240446)
Supplement: S1 Table — Data shown as mean ± standard deviation, median (interquartile range) or n (%) as appropriate. p-value indicates group difference between consolidated cohort and those patients with IS levels exceeding the 95% UPL. IS = indoxyl sulfate; UPL = upper prediction limit; CAKUT = congenital anomalies of the kidney and urinary tract; Post-AKI = chronic kidney disease after acute kidney injury; BMI = body mass index; eGFR = estimated glomerular filtration rate; uPCr = Urinary protein creatinine ratio; SDS = standard deviation score; Non-Ca phosphate binders = non calcium containing phosphate binders; Ca phosphate binders = calcium containing phosphate binders. Countries: GER = Germany; CH = Switzerland; AT = Austria; FR = France; IT = Italy; PT = Portugal; PL = Poland; LT = Lithuania; CZ = Czech Republic; SRB = Serbia; UK = United Kingdom. (PDF) [file pone.0240446.s002.pdf]

S1 Table. Patient characteristics of patients with indoxyl sulfate levels exceeding the 95% upper prediction limit compared to the rest of the cohort (consolidated cohort)

Variable	Total cohort	N=	Consolidated Cohort	N=	Subgroup IS > 95% UPL	N=	P-value
**Age (years)**	12.1 ± 3.3	604	12.2 ± 3.3	559	11.2 ± 3.3	45	**0.05**
**Male sex**	399 (66%)	604	367 (66%)	559	32 (71%)	45	0.46
**Diagnosis**		604		559		45	0.53
**CAKUT**	420 (70%)		390 (70%)		30 (67%)		
**Glomerulopathy**	49 (8%)		46 (8%)		3 (7%)		
**Post-AKI**	27 (5%)		23 (4%)		4 (9%)		
**Tubulointerstitial**	82 (14%)		77 (14%)		5 (11%)		
**Others**	26 (4%)		23 (4%)		3 (7%)		
**Country**		604		559		45	0.07
**GER, CH, AT**	118 (20%)		112 (20%)		6 (13%)		
**FR, IT, PT**	97 (16%)		88 (16%)		9 (20%)		
**PL, LT, CZ, SRB**	53 (9%)		52 (9%)		1 (2%)		
**UK**	35 (6%)		35 (6%)		0 (0%)		
**Turkey**	301 (50%)		272 (49%)		29 (64%)		
**BMI SDS**	0.08 ± 1.30	604	0.08 ± 1.30	559	0.05 ± 1.29	45	0.87
**Systolic blood pressure SDS**	0.81 ± 1.37	604	0.82 ± 1.37	559	0.66 ± 1.45	45	0.46
**eGFR (ml/min)**	26.6 ± 11.2	604	26.6 ± 11.2	559	26.6 ± 10.9	45	0.97
**uPCr (mg/mg)**	1.3 (2.6)	579	1.4 (2.7)	537	1.0 (1.4)	44	0.22
**Serum Albumin (g/l)**	39.0 ± 6.30	604	38.9 ± 6.36	559	38.6 ± 5.67	45	0.70
**Serum phosphorus (mmol/l)**	1.55 ± 0.38	604	1.55 ± 0.37	559	1.65 ± 0.44	45	0.07
**Hemoglobin (g/l)**	11.6 ± 1.65	594	11.7 ± 1.64	550	11.1 ± 1.70	44	**0.03**
**Non-Ca phosphate binders (y)**	25 (4%)	604	24 (4%)	559	1 (2%)	45	0.50
**Ca phosphate binders (y)**	271 (45%)	604	252 (45%)	559	19 (42%)	45	0.71
**Iron supplementation (y)**	267 (44%)	604	246 (44%)	559	21 (47%)	45	0.73
**Antibiotic treatment at baseline (y)**	128 (21%)	604	120 (22%)	559	8 (18%)	45	0.56
**Antibiotic treatment <6 weeks before baseline (y)**	130 (21%)	604	121 (21%)	559	9 (20%)	45	0.80

Data shown as mean ± standard deviation, median (interquartile range) or n (%) as appropriate. P-value indicates group difference between consolidated cohort and those patients with IS levels exceeding the 95% UPL.

IS= indoxyl sulfate; UPL= upper prediction limit; CAKUT= congenital anomalies of the kidney and urinary tract; Post-AKI= chronic kidney disease after acute kidney injury; BMI= body mass index; eGFR= estimated glomerular filtration rate; uPCr= Urinary protein creatinine ratio; SDS= standard deviation score; Non-Ca phosphate binders= non calcium containing phosphate binders; Ca phosphate binders= calcium containing phosphate binders. Countries: GER= Germany; CH= Switzerland; AT= Austria; FR= France; IT= Italy; PT= Portugal; PL= Poland; LT= Lithuania; CZ= Czech Republic; SRB= Serbia; UK= United Kingdom.
