# Supplementary material for: Serum indoxyl sulfate concentrations associate with progression of chronic kidney disease in children
Source: PLoS One. 2020 Oct 27;15(10):e0240446. doi: 10.1371/journal.pone.0240446 (PMC7591021; doi:10.1371/journal.pone.0240446)
Supplement: S2 Table — Diagnostic groups were compared to patients with the diagnosis of CAKUT. Residency was defined as living in Mediterranean or Non-Mediterranean countries. Physical activity was compared to being physically inactive (0 hours). SE = standard error; CAKUT = congenital anomalies of the kidney and urinary tract; Post-AKI = chronic kidney disease after acute kidney injury; BMI = body mass index; eGFR = estimated glomerular filtration rate; Ca-based P binders = calcium-based phosphate binders; uPCr = Urinary protein creatinine ratio; SDS = standard deviation score. (PDF) [file pone.0240446.s003.pdf]

S2 Table. Multivariable linear regression models of variables associated with serum levels of indoxyl sulfate and p-cresyl sulfate in 604 children with CKD at baseline

	**Log indoxyl sulfate**			**Log-p-cresyl sulfate**		
	Estimate ± SE		P-Value	Estimate ± SE		P-Value
Age (years)	-.017	±.016	.274	**.035**	**±.574**	**.008**
Girls	-.146	±.119	.192	-.045	±.013	.629
BMI SDS	.013	±.040	.741	-.036	±.032	.271
**Diagnosis**						
Tubulointerstitial	.165	±.157	.294	-.018	±.129	.888
Glomerulopathy	-.096	±.213	.651	**-.469**	**±.176**	**.008**
Post-AKI	.261	±.266	.326	-.285	±.219	.195
Others	.237	±.261	.367	.419	±.216	.067
**Residency**						
Non-Mediterranean	-.188	±.115	.102	**.350**	**±.095**	**<.001**
**Physical activity**						
1 – 2 hours	-.107	±.163	.510	-.180	±.134	.180
2 – 4 hours	.037	±.179	.835	.162	±.150	.256
> 4 hours	-.037	±.136	.786	**-.226**	**±.112**	**.044**
eGFR (ml/min/1,73m^2)	**-.050**	**±.008**	**<.001**	**-.035**	**±.007**	**<.001**
Serum albumin (g/l)	.001	±.010	.902	**.035**	**±.008**	**<.001**
Urea (mg/dl)	**.013**	**±.004**	**.004**	**.007**	**±.004**	**.038**
Uric acid (mg/dl)	**-.121**	**±.032**	**<.001**	**-.082**	**±.026**	**.002**
Serum phosphorus	.207	±.150	.167	.014	±.123	.911
Log UPCr	.194	±.109	.074	.021	±.047	.652
Ca-based P binders	.001	±.100	.995	.042	±.090	.634
Iron Therapy	.194	±.109	.074	**.190**	**±.090**	**.034**
Antibiotics	-.036	±.135	.790	.034	±.111	.762

Diagnostic groups were compared to patients with the diagnosis of CAKUT. Residency was defined as living in Mediterranean or Non-Mediterranean countries. Physical activity was compared to being physically inactive (0 hours).

SE= standard error; CAKUT= congenital anomalies of the kidney and urinary tract; Post-AKI= chronic kidney disease after acute kidney injury; BMI= body mass index; eGFR= estimated glomerular filtration rate; Ca-based P binders= calcium-based phosphate binders; uPCr= Urinary protein creatinine ratio; SDS= standard deviation score.
