# Supplementary material for: Serum indoxyl sulfate concentrations associate with progression of chronic kidney disease in children
Source: PLoS One. 2020 Oct 27;15(10):e0240446. doi: 10.1371/journal.pone.0240446 (PMC7591021; doi:10.1371/journal.pone.0240446)
Supplement: S3 Table — Survival analysis using the PHREG SAS procedure. If the event occurred between two visits the later of the two visits was taken as occurrence of the event instead of using linear interpolation. Model 0 represents a Cox model with sex, age, diagnosis, region of residence, body mass index SDS, blood pressure SDS, estimated glomerular filtration rate, urinary protein creatinine ratio, hemoglobin, serum phosphorus and albumin as covariates, calculated either for the total cohort (n = 569) or for the consolidated cohort (excluding patients with IS levels exceeding the 95% upper prediction limit, n = 526). Model 1a includes serum IS, 1b IS quartiles, 1c serum pCS and 1d serum pCS quartiles as a covariate to model 0. Models 1e and 1 f include serum IS and serum IS quartiles respectively as covariates to model 0 in the consolidated cohort. IS and pCS are used as logarithmic values in all models. For all variables associated with renal survival (model 0, total cohort) please refer to Table 3. IS = indoxyl sulfate; pCS = p-cresyl sulfate; Q = quartiles of serum IS and pCS levels, respectively. (PDF) [file pone.0240446.s004.pdf]

**S3 Table. Multivariable Cox proportional hazard analysis for the association of IS and pCS serum levels with renal survival (sensitivity analysis by taking the later visit if the event occurred between two visits)**

| | Parameter | p-value | Hazard Ratio | 95% Hazard Ratio Confidence Intervall | |
|---|---|---|---|---|---|
| **Model 0 total cohort** | --- | | --- | --- | --- |
| Model 1a | Log IS | 0.106 | 1.07 | 0.98 | 1.17 |
| Model 1b | Q2: 2.9-5.3 | 0.443 | 1.15 | 0.80 | 1.64 |
| | Q3: 5.3-11.6 | 0.416 | 0.86 | 0.59 | 1.26 |
| | Q4: >11.6 | **0.010** | **1.64** | **1.13** | **2.38** |
| Model 1c | Log pCS | 0.634 | 0.97 | 0.87 | 1.09 |
| Model 1d | Q2: 7.9-17.2 | 0.216 | 0.80 | 0.58 | 1.13 |
| | Q3: 17.2-29.6 | 0.308 | 0.84 | 0.60 | 1.12 |
| | Q4: >29.6 | 0.603 | 0.91 | 0.65 | 1.30 |
| **Model 0 consolidated cohort** | --- | | --- | --- | --- |
| Model 1e | Log IS | **0.007** | **1.24** | **1.06** | **1.45** |
| Model 1f | Q2: 2.9-5.3 | 0.404 | 1.16 | 0.81 | 1.67 |
| | Q3: 5.3-11.6 | 0.565 | 0.89 | 0.61 | 1.31 |
| | Q4: >11.6 | **0.002** | **1.94** | **1.28** | **2.93** |

Survival analysis using the PHREG SAS procedure. If the event occurred between two visits the later of the two visits was taken as occurrence of the event instead of using linear interpolation. Model 0 represents a Cox model with sex, age, diagnosis, region of residence, body mass index SDS, blood pressure SDS, estimated glomerular filtration rate, urinary protein creatinine ratio, hemoglobin, serum phosphorus and albumin as covariates, calculated either for the total cohort (n=569) or for the consolidated cohort (excluding patients with IS levels exceeding the 95% upper prediction limit, n=526). Model 1a includes serum IS, 1b IS quartiles, 1c serum pCS and 1d serum pCS quartiles as a covariate to model 0. Models 1e and 1 f include serum IS and serum IS quartiles respectively as covariates to model 0 in the consolidated cohort. IS and pCS are used as logarithmic values in all models. For all variables associated with renal survival (model 0, total cohort) please refer to Table 3. IS= indoxyl sulfate; pCS= p-cresyl sulfate; Q= quartiles of serum IS and pCS levels, respectively.
