# Supplementary material for: Serum indoxyl sulfate concentrations associate with progression of chronic kidney disease in children
Source: PLoS One. 2020 Oct 27;15(10):e0240446. doi: 10.1371/journal.pone.0240446 (PMC7591021; doi:10.1371/journal.pone.0240446)
Supplement: S4 Table — Survival analysis for interval-censored data using the ICPHREG SAS procedure. Model 0 represents a Cox model with sex, age, diagnosis, region of residence, body mass index SDS, blood pressure SDS, estimated glomerular filtration rate, urinary protein creatinine ratio, hemoglobin, serum phosphorus and albumin as covariates, calculated either for the total cohort (n = 569) or for the consolidated cohort (excluding patients with IS levels exceeding the 95% upper prediction limit, n = 526). Model 1a includes serum IS, 1b IS quartiles, 1c serum pCS and 1d serum pCS quartiles as a covariate to model 0. Models 1e and 1 f include serum IS and serum IS quartiles respectively as covariates to model 0 in the consolidated cohort. IS and pCS are used as logarithmic values in all models. For all variables associated with renal survival (model 0, total cohort) please refer to Table 3. IS = indoxyl sulfate; pCS = p-cresyl sulfate; Q = quartiles of serum IS and pCS levels, respectively. (PDF) [file pone.0240446.s005.pdf]

S4 Table. Multivariable Cox proportional hazard analysis for the association of IS and pCS serum levels with renal survival (sensitivity analysis by considering interval censoring if the event occurred between two visits)

	Parameter	p-value	Hazard Ratio	95% Hazard Ratio Confidence Intervall	
**Model 0 total cohort**	---		---	---	---
Model 1a	Log IS	0.160	1.07	0.97	1.16
Model 1b	Q2: 2.9-5.3	0.323	1.20	0.84	1.71
	Q3: 5.3-11.6	0.734	0.94	0.64	1.37
	Q4: >11.6	**0.022**	**1.56**	**1.06**	**2.30**
Model 1c	Log pCS	0.361	0.95	0.84	1.07
Model 1d	Q2: 7.9-17.2	0.312	0.84	0.60	1.18
	Q3: 17.2-29.6	0.468	0.88	0.62	1.25
	Q4: >29.6	0.392	0.85	0.59	1.23
**Model 0 consolidated cohort**	---		---	---	---
Model 1e	Log IS	**0.025**	**1.20**	**1.02**	**1.41**
Model 1f	Q2: 2.9-5.3	0.334	1.19	0.83	1.72
	Q3: 5.3-11.6	0.817	0.96	0.65	1.41
	Q4: >11.6	**0.008**	**1.79**	**1.17**	**2.75**

Survival analysis for interval-censored data using the ICPHREG SAS procedure. Model 0 represents a Cox model with sex, age, diagnosis, region of residence, body mass index SDS, blood pressure SDS, estimated glomerular filtration rate, urinary protein creatinine ratio, hemoglobin, serum phosphorus and albumin as covariates, calculated either for the total cohort (n=569) or for the consolidated cohort (excluding patients with IS levels exceeding the 95% upper prediction limit, n=526). Model 1a includes serum IS, 1b IS quartiles, 1c serum pCS and 1d serum pCS quartiles as a covariate to model 0. Models 1e and 1 f include serum IS and serum IS quartiles respectively as covariates to model 0 in the consolidated cohort. IS and pCS are used as logarithmic values in all models. For all variables associated with renal survival (model 0, total cohort) please refer to Table 3. IS= indoxyl sulfate; pCS= p-cresyl sulfate; Q= quartiles of serum IS and pCS levels, respectively.
