# Supplementary material for: Serum indoxyl sulfate concentrations associate with progression of chronic kidney disease in children
Source: PLoS One. 2020 Oct 27;15(10):e0240446. doi: 10.1371/journal.pone.0240446 (PMC7591021; doi:10.1371/journal.pone.0240446)
Supplement: S5 Table — CAKUT = congenital anomalies of the kidney and urinary tract. (PDF) [file pone.0240446.s006.pdf]

S5 Table. Antibiotic therapy at baseline and within 6 weeks before baseline stratified by diagnosis group

	All	CAKUT	Glomerulopathy	Others	P-value
Antibiotics at baseline					
- no	476 (78.8%)	298 (71.0%)	46 (93.9%)	132 (97.8%)	**<.001**
- yes	128 (21.2%)	122 (29.0%)	3 (6.1%)	3 (2.2%)	
Antibiotics <6 weeks before baseline					
- no	474 (78.5%)	297 (70.7%)	45 (91.8%)	132 (97.8%)	**<.001**
- yes	130 (21.5%)	123 (29.3%)	4 (8.2%)	3 (2.2%)	

CAKUT= congenital anomalies of the kidney and urinary tract.
